# Risk factors of health care–associated infection in elderly patients: a retrospective cohort study performed at a tertiary hospital in China

**DOI:** 10.1186/s12877-019-1208-x

**Published:** 2019-07-19

**Authors:** Xia Zhao, Lihong Wang, Nan Wei, Jingli Zhang, Wenhui Ma, Huijie Zhao, Xu Han

**Affiliations:** 1 Hospital Infection Management Division, Xuanwu Hospital, Capital Medical University, No.45 Changchun Street, Xicheng District, Beijing, 100053 China; 2 School of Health Management and Education, Capital Medical University, Beijing, China

**Keywords:** Health-care associated infection, Elderly, Risk factors, Retrospective cohort study

## Abstract

**Background:**

The elderly inpatients are in high risk of suffering health-care associated infection (HAI). The study aimed to analyze the risk factors of health-care associated infection (HAI) in elderly hospitalized patients to prevent it and improve the recovery rate of elderly patients.

**Methods:**

The study was a Retrospective Cohort Study based on a 3-year surveillance in elderly inpatients in a large tertiary hospital in China. A retrospective review of the elderly inpatients ≥60 years with or without HAI were conducted. Binary multivariable logistic regression was used to evaluate the potential association between HAI and risk factors.

**Results:**

We investigated a total of 60,332 subjects aged 60 years old or above. The incidence of HAI in elderly was 2.62%. With adjustment for some factors, advanced age, hospital days before HAI, intensive care unit (ICU) admission, use of ventilator, central line catheter or urinary catheter and cerebral hemorrhage, cerebral infarction, brain neoplasms, diabetes mellitus, coronary artery disease, malignant tumor and malignant hematonosis had significantly increased odds ratios (OR) of suffering from HAI compared with the control group but body weight and operation decreased OR.

**Conclusion:**

Our findings suggested that advanced age, accompanied by some neurological and chronic noncommunicable diseases, hospital days before HAI, ICU admission, and use of devices were risk factors of suffering HAI in the elderly but the body weight and operation were the potential protective factors in this sample.

## Background

Health-care associated infections (HAIs) which occur in patients under medical care in hospital or other health care facility is a notable public health concern. They place an enormous burden on poor prognosis, increased mortality, prolong hospitalization, and increased health-care costs. Every day, roughly one in 25 patients in the USA contracts at least one infection during their hospital care—an alarming statistic that is unacceptable in view of the fact that health care-associated infections (HAIs) are mostly preventable [1]. However, HAIs are preventable with adoption of recognized preventive measures. Over the past decade, a downward trend in health care-associated infections has occurred. A Survey of a total 12,299 patients in 199 hospitals showed that fewer patients had health care-associated infections in 2015 (394 patients [3.2%; 95% confidence interval {CI}, 2.9 to 3.5]) than in 2011 (452 [4.0%; 95% CI, 3.7 to 4.4]) (*P* < 0.001), largely owing to reductions in the prevalence of surgical-site and urinary tract infections [2].

The risk factors of HAI concluded extrinsic factors such as invasive procedures, medication and stays in risk units and the conditions of the patient’s own condition such as age, sex, body weight, intrinsic comorbidities and immunological factors on the basis of some previous reports and clinical experience of senior doctors. A systematic review and meta-analysis about risk factors for HAI in hospitalized adults showed that the major risk factors independently associated with HAIs were diabetes mellitus, immunosuppression, body temperature, surgery time in minutes, reoperation, cephalosporin exposure, days of exposure to central venous catheter, intensive care unit (ICU) admission, ICU stay in days, and mechanical ventilation [3].

The elderly are vulnerable to infections due to their reduced immunological competence and complication of chronic illness [4–7]. China is one of the most advanced aging society and aging societies have increased the number of elderly inpatients. In order to take effective intervention to prevent HAIs in elderly, the first step is to find risk factors and identify patients at higher risk of HAI. Published research papers revealed some risk factors for some special sites of HAIs such as surgical site infection (SSI), urinary tract infections, HAIs occured frequently in neurological ICU, etc. [8–11]. But there were few research focused on risk factors of HAIs in elderly. This study aimed to identify risk factors (RFs) present on admission and acquired during inpatient stay which could be associated with higher risk of acquiring HAI in elderly. Then we can formulate intervention strategies in the light of high-risk patients and risk factors to reduce the incidence of HAIs and improve the recovery rate of elderly patients.

## Methods

### Study design and data collection

The study was a Retrospective Cohort Study based on a 3-year surveillance in elderly inpatients in a large tertiary hospital with 1147 beds in Beijing, China. A retrospective review of the elderly inpatients ≥60 years old with or without HAI were conducted and there was a cohort design. A total of 60,332 subjects (≥60 years old) who had been hospitalized from January 1, 2015 to December 31, 2017 were admitted and the subjects with less than 2 days or more than 60 days of hospitalization were excluded.

HAIs were defined as infections occurred 48 h after admission in inpatients. All HAIs that occurred in their hospitalized stay were identified by infection control practitioners and doctors according to the definitions published by Ministry of Health, the People’s Republic of China in 2001 [12].

We collected 5 categories including 22 kinds of information of each participant including demographic characteristics, hospitalization days, diagnoses, operations, and specific device days using an automatic online HAI surveillance system named real-time nosocomial infection surveillance system (RT-NISS, VERSION:12.8.2.1). The RT-NISS is an integrated online HAI surveillance system which automatically download data from other information systems including the Hospital Information System (HIS), Electronic Medical Record (EMR), Laboratory Information System (LIS), Picture Archiving and Communication System (PACS), Mobile Nursing Information System (MNIS) and Anesthesia Operation System (AOS) to record clinical information of the inpatients. And then the RT-NISS screen the potential HAIs according to the Chinese NI diagnosis criterion published by the Ministry of Public Health in 2001 automatically which pre-input to the system by the algorithm of microbiological reports, radiology information, serological and molecular testing, antibiotic usage and fever history of the patients [13].

All collected data was checked by the infection control team and removed invalid data. Then All the potential HAIs were identified by infection control practitioners and doctors according to the definitions published by Ministry of Health, the People’s Republic of China in 2001. So the data we collected was validity and reliability.

### Statistical analysis

The data was consisted of measurement data and numeration data and was analyzed using the software SPSS 13.0. All the continuous variables were presented as means ± standard deviation (M ± s.d.) and the significance of the difference between the two groups was analyzed by using Independent-Samples t test. The category variables were analyzed by using Chi-square or Fisher’s exact test. Binary multivariable logistic regression was performed to evaluate the potential association between the variables and HAI and the odds ratio (OR) and 95% confidence interval (CI) were calculated at the same time. Statistical testing was performed at the conventional 2-tailed α = 0.05.

## Results

### The characteristics of the study elderly inpatients with and without HAI

There were 60,332 subjects, including 1580(2.62%) subjects with HAIs. The median age is 69 (range from 60 to 104) and there were 33280(55.16%) males and 27052(44.84%) females. Table 1 and Table 2 show the characteristics of the study elderly inpatients with and withoutHAI in univariate analysis.

**Table 1:** Hospital days and body weight of the subjects with and without HAI in univariate analysis, continuous variables, aged 60+, in Beijing, China

	Control group *N* = 58752	HAI group *N* = 1580	*t*	*P*
	M ± s.d	M ± s.d		
Total hospital days (days)	9.16 ± 5.84	21.81 ± 10.45	47.93	< 0.001
Hospital days before HAI (days)	9.16 ± 5.84	10.31 ± 6.95	6.37	< 0.001
Body weight (km)	66.92 ± 11.69	64.46 ± 11.59	6.21	< 0.001

**Table 2:** Age, sex, operations, ICU admission, using devices and chronic diseases of the subjects with and without HAI in univariate analysis, category variables, aged 60+, in Beijing, China (As a retrospetive study, there were some missing values about chronic diseases)

Variables		No. of subjects	No. of HAIs	Incidence (%)	*χ* ^*2*^	*P*
Age	60~69y	32156	637	1.98	241.76	< 0.001
	70~79y	18203	462	2.54		
	≥80y	9973	481	4.82		
Sex	Male	33280	909	2.73	3.69	0.06
	Female	27052	671	2.48		
Operation	No	32627	947	2.90	22.42	< 0.001
	Yes	27705	633	2.28		
ICU days	d = 0	51980	911	1.75	3625.18	< 0.001
	d < 2	1742	46	2.64		
	2 ≤ d < 7	4120	159	3.86		
	7 ≤ d < 14	1472	154	10.46		
	d ≥ 14	1018	310	30.45		
Using ventilator	No	58478	1191	2.04	2529.06	< 0.001
	Yes	1854	389	2.10		
Using central line catheter	No	56338	1015	1.80	2228.59	< 0.001
	Yes	3994	565	14.15		
Using urinary catheter	No	43956	626	1.42	906.33	< 0.001
	Yes	16376	954	5.82		
Cerebral hemorrhage	No	56866	1359	2.39	122.02	< 0.001
	Yes	2770	160	5.78		
Cerebral infarction	No	39857	871	2.19	63.38	< 0.001
	Yes	19779	648	3.28		
Brain neoplasms	No	59192	1496	2.53	12.49	< 0.001
	Yes	444	23	5.18		
Hypertension	No	25586	590	2.31	10.50	< 0.001
	Yes	34050	929	2.73		
Hyperlipidemia	No	51189	1330	2.60	3.84	0.05
	Yes	8453	189	2.23		
Diabetes mellitus	No	40751	985	2.42	8.76	< 0.001
	Yes	18885	534	2.83		
Coronary artery disease	No	41284	825	2.00	162.76	< 0.001
	Yes	18352	694	3.78		
COPD	No	57510	1438	2.50	14.16	< 0.001
	Yes	2126	81	3.81		
Malignant tumor	No	49227	1203	2.44	12.13	< 0.001
	Yes	10409	316	3.04		
Malignant hematonosis	No	57123	1229	2.15	854.76	< 0.001
	Yes	2513	290	11.53		
Osteoarthropathy	No	56429	1435	2.54	0.07	0.79
	Yes	3207	84	2.62		
Gynecological diseases	No	58860	1510	2.56	6.10	0.020
	Yes	776	9	1.16		

### The potential association between risk factors and HAI

The results of the multivariable logistic regression analysis suggest a significant association between HAI and some relevant factors. With adjustment for some factors, age, hospital days before HAI, ICU admission, use of ventilator, central line catheter or urinary catheter and some neurological and chronic noncommunicable diseases including cerebral hemorrhage, cerebral infarction, brain neoplasms, diabetes mellitus, coronary artery disease, malignant tumor and malignant hematonosis had significantly increased odds ratios (OR) of suffering from HAI compared with the control group but body weight and operation decreased OR (Table 3).

**Table 3:** Adjusted odds ratios for the association between HAI and risk factors among inpatients aged 60+, Beijing, China

	β	Wals *χ2*	OR	95% CI.		*P*
				Lower limit	Upper limit	
Age	.204	16.168	1.226	1.110	1.355	< 0.001
Body weight	−.015	21.077	.985	.979	.991	< 0.001
Hospital days before HAI	−.026	16.718	1.138	1.131	1.145	< 0.001
Operation	−.370	14.642	.691	.572	.835	< 0.001
ICU admission	.388	11.606	1.474	1.179	1.842	.001
Using ventilator	.624	16.419	1.867	1.380	2.525	< 0.001
Using central line catheter	1.350	159.015	3.856	3.126	4.755	< 0.001
Using urinary catheter	1.047	102.414	2.848	2.325	3.488	< 0.001
Cerebral hemorrhage	.329	5.662	1.389	1.060	1.821	.017
Cerebral infarction	.370	20.308	1.448	1.233	1.701	< 0.001
Brain neoplasms	.359	12.419	1.432	1.173	1.748	< 0.001
Diabetes mellitus	.181	5.196	1.198	1.026	1.400	.023
Coronary artery disease	.166	3.968	1.180	1.003	1.389	.046
Malignant tumor	.162	5.946	1.176	1.032	1.339	.015
Malignant hematonosis	1.613	244.942	5.018	4.100	6.141	< 0.001

Notes: Binary multivariable logistic regression was performed. Statistical testing was performed at the conventional 2-tailed α = 0.05.*OR* Odds ratio

*CI* confidence interval

Only significant predictors are presented. Variables adjusted in the model include age, body weight, hospital days before HAI, operation, ICU admission, use of ventilator, central line catheter and urinary catheter and some neurological and chronic noncommunicable diseases (including cerebral hemorrhage, cerebral infarction, brain neoplasms, hypertension, diabetes mellitus, coronary artery disease, COPD, malignant tumor, malignant hematonosis and gynecological diseases)

## Discussion

This survey based on the retrospective cohort data showed that the incidence of HAI in elderly sample aged 60+ was 2.62% which was lower than many other reports but consistent with our previous study and some similar studies in the same region. Our study about the characters of HAI in venerable elderly hospitalized patients which indicated the incidence of HAI in venerable elderly (aged 70+) were significantly higher than those under 70 years old (3.38% vs 1.45%, P<0.05) [14]. The investigation of the HAIs incidence in elderly hospitalized patients at another hospital in Beijing, China, reported that the HAI incidence in patients aged ≥60y was significant higher than that in all inpatients (2.57% vs 1.84%,χ^2^ = 70.493, *P* < 0.05) [15]. A survey of the prevalence of HAIs in older people in acute care hospitals in Scotland found a linear relationship between prevalence of HAI and increasing age which described a pooled prevalence of HAIs in patients less than 65y and more than 65y (7.37% vs 11.13%, *P* < 0.05) [16]. The low incidence of HAI in this sample probably due to the effective prevention or specific diseases in the hospital. This hospital is excellent at diagnosis and therapy of neuroscience and geriatrics, so the nursing care of elderly patients is professional and the average length of hospital stay is shorten which contribute to the low incidence of HAI. On the other hand, the surveillance definition of HAIs is not entirely consistent in different research which lead to the difference in the result of the incidence of HAI.

The present binary multivariable logistic regression with adjustment for some factors showed that age, hospital days before HAI, intensive care unit (ICU) admission, use of ventilator, central line catheter or urinary catheter and cerebral hemorrhage, cerebral infarction, brain neoplasms, diabetes mellitus, coronary artery disease, malignant tumor and malignant hematonosis were the dependent risk factors of suffering from HAIs based on this elderly sample. These results were similar to the reported conclusion based on adults about the risk factors of HAIs. Length of ICU stay, diabetes and COPD were risk factors in a study of risk factors and epidemiology of HAI from an intensive care unit in Northern India [17]. Published studies observed some intrinsic risk factors such as age > 65 years, terminal incurable disease, gastrointestinal diseases and the presence of > 2 underlying diseases [18, 19]. Other reports showed some extrinsic risk factors such as ICU admission, previous antibiotic use, invasive mechanical ventilation, hospitalization time [20, 21]. So for elderly patients, the unnecessary hospital day and device using should be cut off to prevent HAIs. And for the elderly patients accompanied by some neurological and chronic noncommunicable diseases, we should enhance the intervention to reduce the risk of suffering from HAIs.

On the opposite side of the above results, body weight exhibited a protective effect for elderly inpatients to prevent HAIs. Published studies suggested that weight loss should be added to the list of risk factors for some special infections such as invasive aspergillosis, tuberculosis and other chronic infection [22, 23]. Weight loss reflects a failure of dietary intake to maintain an adequate nutritional status and results in immuno-compromised to be vulnerable to infections. So the elderly inpatients should achieve weight maintenance and have a healthier body mass and composition to prevent HAIs.

Another result of this study was that operation significantly decreased the OR of suffering from HAIs in this elderly population. Operations play an important role in SSIs which is one critical category of HAIs and there were many studies on risk factors for SSIs occurring different kinds of surgical procedures. The relationship between patient age and the risk of SSI is not consistently reported in the literature, with numerous studies that implicating advanced age as a risk factor for SSI, and numerous studies finding no such association. Although SSI is one of the complications causing poor prognosis postoperative patients, the incidence is reported to vary from 0.1 to 50% [24, 25]. But general incidence of SSI of the most operations is lower than the common HAIs such as pneumonia, urinary tract infection and blood stream infection, especially in elderly. Our previous study showed that the top 3 sites of HAIs in elderly were lower respiratory tract infections, urinary system infections and blood stream infections and the ratio was 41.62, 15.44 and 9.60% respectively, but the ratio of SSIs was only 2.26% [14]. Furthermore, the physical conditions of the elderly inpatients for the purpose of surgical treatment maybe were better than those suffering from many kinds of medical diseases, so they would not likely to be infected. This is probably the potential reason to explain this result in the present real world elderly population.

There were some limitations in the present study including the lack of analysis of special risk factors of special infections such as pneumonia, urinary infection, bloodstream infection and SSI in elderly. There are probably some special risk factors in a special infection attribute to the characteristics of different infection sites. We will think highly of this limitations and make an effort to reveal in our following study.

## Conclusion

Our findings suggested that the risk factors of HAI in elderly included extrinsic factors such as advanced age, accompanied by some neurological and chronic noncommunicable diseases and intrinsic factors such as hospital days before HAI, ICU admission, and use of devices. But body weight indicated a potential protective effect on elderly population to prevent HAI. On the other hand, operation was not the risk factor of HAI and the operative elderly patients take lower risk of suffering HAI in this elderly population.

## Data Availability

The datasets generated and/or analyzed during the current study are not publicly available due the data copyright protection of the author’s institute, but are available from the corresponding author on reasonable request.

## Abbreviations

CAUTI: Catheter-associated urinary tract infection
CI: Confidence intervals
CLABSI: Central line-associated bloodstream infections
HAI: Healthcare associated infections
ICU: Intensive care unit
NISS: Infection surveillance system
VAP: Ventilator-associated pneumonia
